# Libman-Sacks Endocarditis as a Noninfectious Cardiac Complication of Acute Promyelocytic Leukemia in an Adolescent

**DOI:** 10.1016/j.case.2026.03.004

**Published:** 2026-05-04

**Authors:** Nabil Nafez Alhayek, Andrew Cave, Khaled Shihabi, Mohanageetha Ardhanari, Arshid Mir

**Affiliations:** a Department of Pediatrics, University of Oklahoma Health Sciences Center, Oklahoma City, Oklahoma; b University of Oklahoma College of Medicine, Oklahoma City, Oklahoma; c Section of Pediatric Cardiology, University of Oklahoma Health Sciences Center, Oklahoma City, Oklahoma

**Keywords:** Libman-Sacks endocarditis, Acute promyelocytic leukemia, Pediatrics, Nonbacterial thrombotic endocarditis

## Abstract

• LSE is a very rare disorder in the pediatric age group.
• Malignancy-associated LSE has not been previously reported in pediatrics.
• LSE may resemble IE in children.
• Early anticoagulation and steroids stabilized leukemia-associated LSE.

## Graphical abstract

**Figure:**
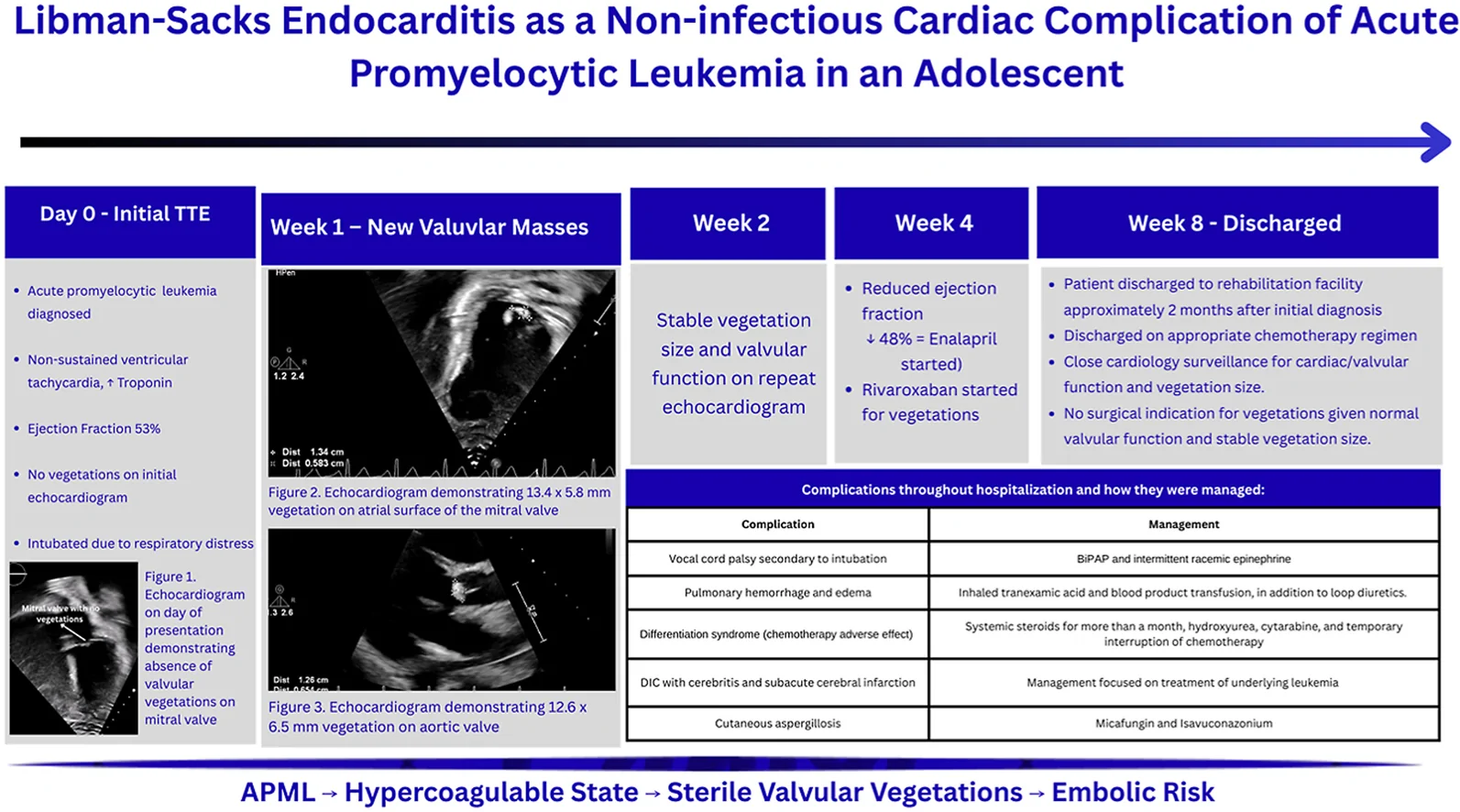

## Introduction

Libman-Sacks endocarditis (LSE), or nonbacterial thrombotic endocarditis, is characterized by sterile, noninfectious vegetations that occur on cardiac valves, most commonly involving the mitral valve (MV) and aortic valve (AV).^1^ LSE has long been associated with systemic autoimmune diseases such as systemic lupus erythematosus (SLE) and antiphospholipid syndrome, where chronic inflammation and a tendency to form blood clots play a key role in its development.^2^^,^^3^ Although malignancy-associated LSE is becoming more recognized in adults,^4^^,^^5^ it remains rarely reported in children. Recognizing LSE in children without an autoimmune background can be especially challenging.^3^^,^^6^ Given their shared predilection for the MV and AV, nonspecific echocardiographic findings, and increased risk for thromboembolic events, LSE may resemble infective endocarditis (IE) on imaging.^7^ That makes early recognition and the correct diagnosis essential, not only to avoid unnecessary antibiotic exposure but also to start anticoagulation and systemic steroids when appropriate to reduce the risk for embolic complications. Here, we describe a unique case of a teenager newly diagnosed with acute promyelocytic leukemia (APML) who developed LSE early in the disease course.

## Case Presentation

A previously healthy 14-year-old boy presented to the emergency department with chief symptoms of fever, tachycardia, shortness of breath, and progressively worsening fatigue. Pertinent vital signs upon arrival were a body temperature of 39.1 °C, a heart rate of 157 beats/min, a respiratory rate of 28 breaths/min, and a body mass index of 39 kg/m^2^. Physical examination was pertinent for obesity, tachycardia with no murmurs and normal rhythm, tachypnea with respiratory distress, bilateral rhonchi, and pale skin. Initial laboratory evaluation was notable for leukopenia (white blood cell count 1.4 × 10^9^/L), neutropenia (neutrophil count 110 cells/μL), severe anemia (hemoglobin 3.7 g/dL, hematocrit 11%), and thrombocytopenia (platelet count 18 × 10^9^/L). Electrolyte abnormalities included mild hyponatremia (sodium 128 mmol/L) and hypochloremia (chloride 96 mmol/L). Acid-base analysis demonstrated high–anion gap metabolic acidosis (venous pH 7.16, bicarbonate 12 mmol/L, anion gap 20 mmol/L) and hyperglycemia (glucose 232 mg/dL). Inflammatory markers were elevated, with C-reactive protein at 62 mg/L and procalcitonin at 1.3 ng/mL. Additional abnormalities included hyperuricemia (uric acid 8.7 mg/dL), coagulopathy (prothrombin time 23 s, international normalized ratio 2.1, D-dimer 1,050 ng/mL), and elevated lactate dehydrogenase (322 U/L). Cardiac biomarkers were notable for elevated troponin (379 ng/L) and pro–brain natriuretic peptide (766 pg/mL). Chest radiography showed bilateral infiltrates. While undergoing workup, the patient developed monomorphic nonsustained ventricular tachycardia that was treated with intravenous lidocaine and amiodarone infusion. Initial transthoracic echocardiography (TTE) upon admission showed a structurally normal heart with mildly reduced global left ventricular (LV) systolic function (LV ejection fraction [LVEF] 54%). The patient was intubated shortly after arrival given respiratory distress. The patient was started on broad-spectrum antibiotics given their leukopenia and had also required multiple blood product transfusions to treat their coagulopathy.

This presentation and the associated laboratory findings prompted the evaluation for an underlying neoplastic process. Further workup including flow cytometry, bone marrow biopsy, and fluorescence in situ hybridization yielded a diagnosis of APML. The patient was started on all-trans retinoic acid and arsenic chemotherapeutic regimen.

The patient’s hospital course was complex and marked by multiple critical complications. These included prolonged mechanical ventilation for >1 month with subsequent vocal cord palsy, pulmonary hemorrhage and edema, differentiation syndrome, leukemic retinopathy, disseminated intravascular coagulation (DIC), cerebritis with subacute cerebral infarctions, cutaneous aspergillosis, and nonsustained ventricular tachycardia.

Vocal cord palsy manifested as stridor and increased work of breathing upon extubation and was attributed to prolonged intubation. Management included systemic dexamethasone, bilevel positive airway pressure, and intermittent racemic epinephrine.

Pulmonary hemorrhage presented as persistent bloody secretions in the endotracheal tube and was treated with inhaled tranexamic acid and blood product transfusions. Pulmonary edema and fluid overload manifested as increased ventilatory requirements and were supported by bilateral pulmonary haziness and congestion on chest radiographs. These were managed with loop diuretics, including bumetanide and furosemide.

Differentiation syndrome manifested as respiratory distress, fever, hypoxia, pulmonary infiltrates, and leukocytosis. Management consisted of systemic dexamethasone for >1 month, cytoreduction with hydroxyurea and cytarabine, and temporary interruption of chemotherapy.

DIC manifested as subacute cerebral and splenic infarctions. Both DIC and leukemic retinopathy were attributed to the underlying APML, and management focused primarily on treatment of the malignancy itself.

Cutaneous aspergillosis presented as two violaceous papules on the right forearm, confirmed by skin biopsy, and was treated with systemic antifungal therapy using micafungin and isavuconazonium. Leukemic retinopathy manifested as mild worsening of vision and was monitored conservatively.

Lidocaine was subsequently discontinued, and the patient remained on amiodarone therapy for approximately 1 month before being transitioned to oral atenolol, with no recurrence of sustained arrhythmias.

**Figure 1:**
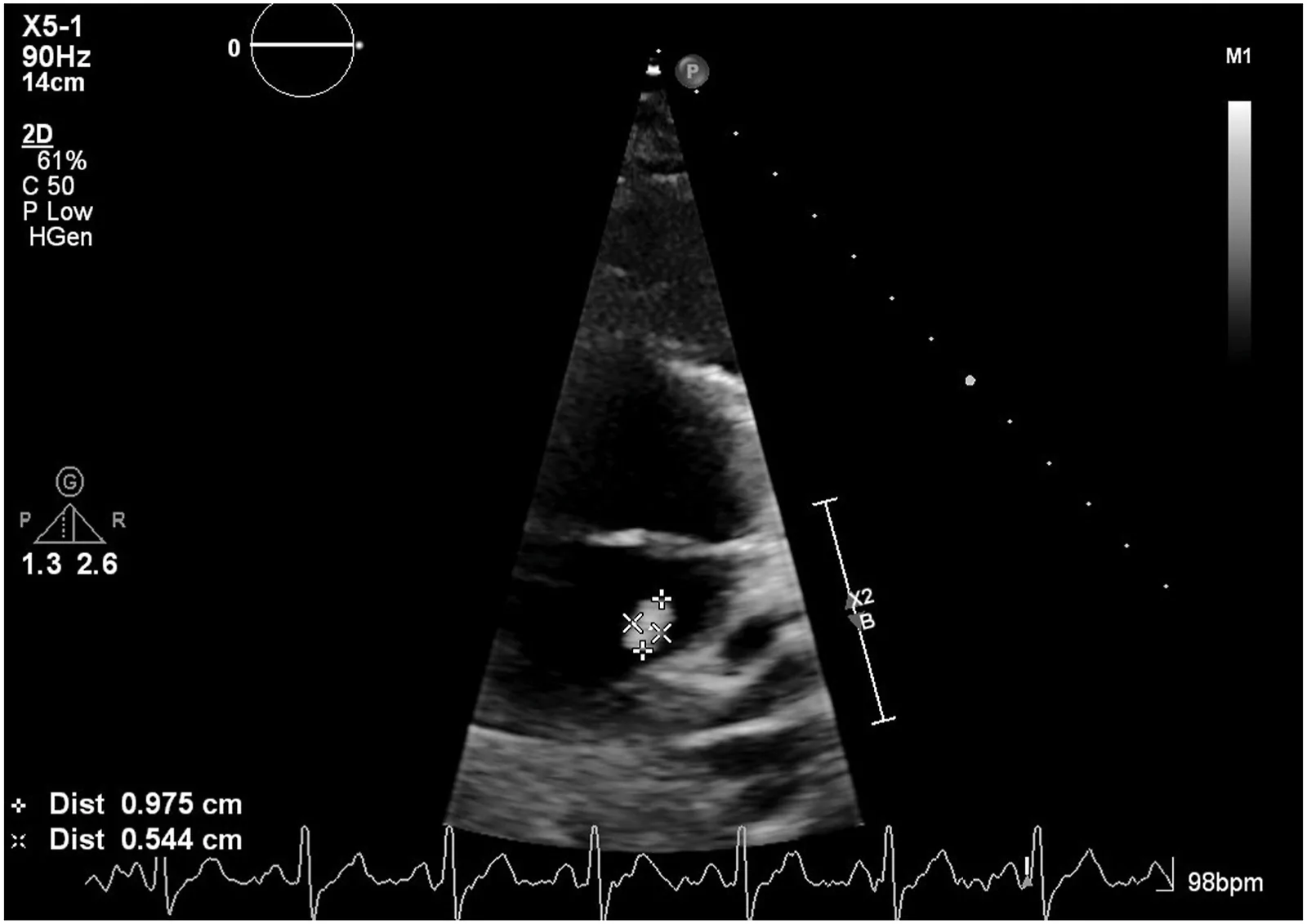
Two-dimensional TTE, zoomed parasternal long-axis systolic view, demonstrates an irregular, echogenic mass (9.8 × 5.4 mm) attached to the atrial surface of the MV leaflet, consistent with a vegetation.

**Figure 2:**
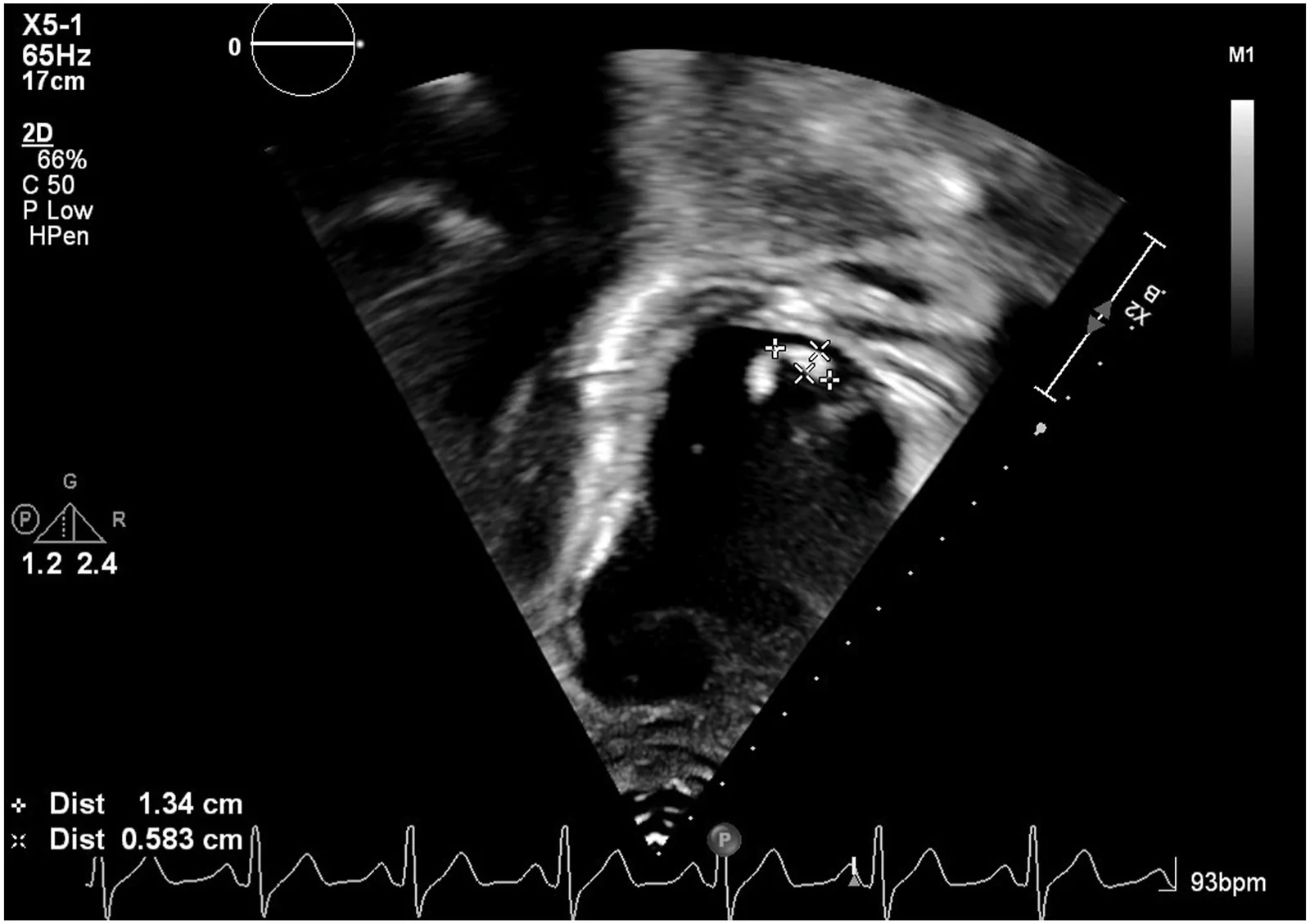
Two-dimensional TTE, apical two-chamber mid-diastolic view, demonstrates an irregular, echogenic mass (13.4 × 5.8 mm) on the atrial surface of the MV, consistent with a vegetation.

**Figure 3:**
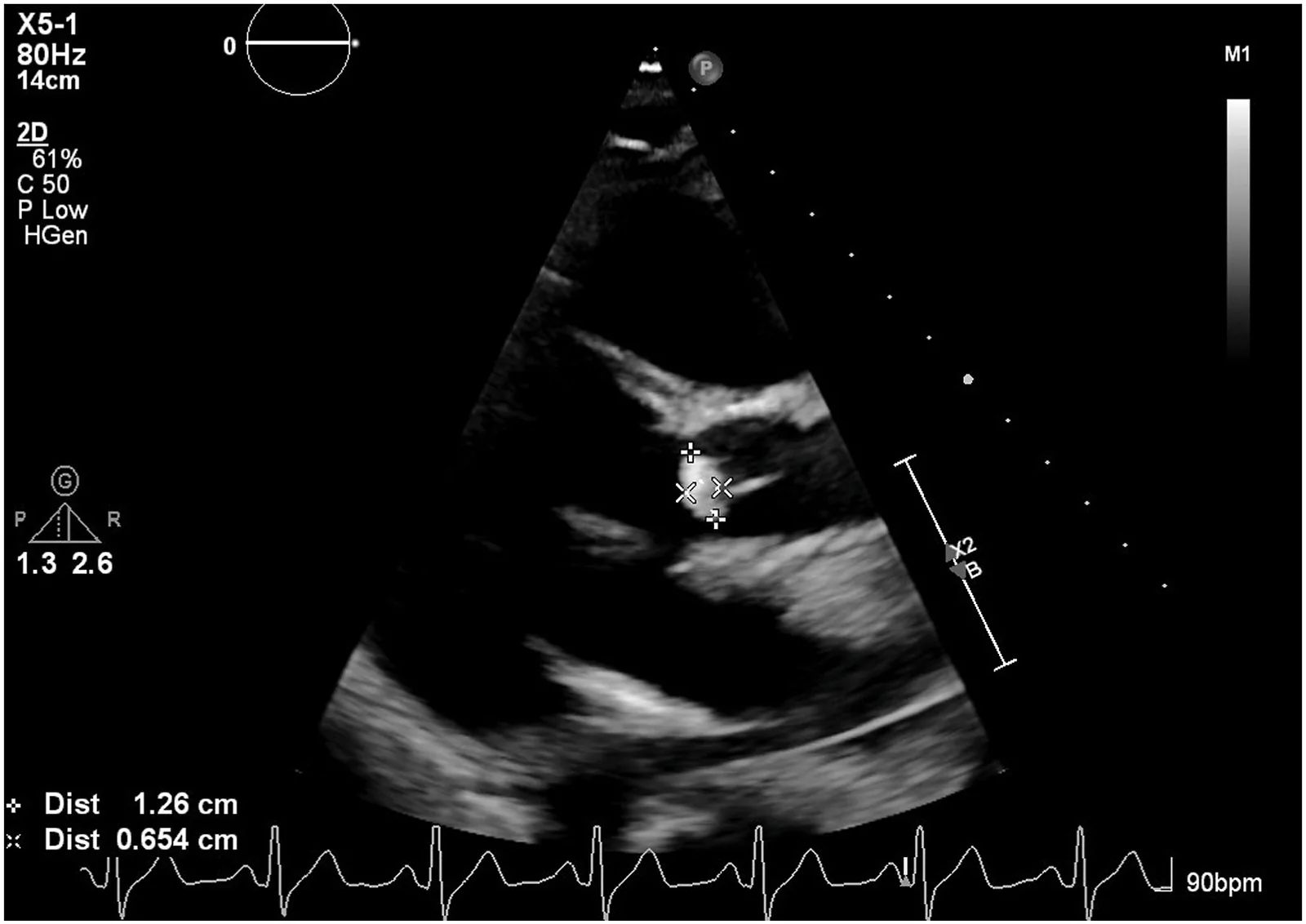
Two-dimensional TTE, parasternal long-axis diastolic view, demonstrates an irregular, sessile, echogenic mass (12.6 × 6.5 mm) attached to the ventricular surface of the noncoronary AV leaflet, consistent with a vegetation.

Troponin levels trended down from 379 to 196.5 ng/L over the first 4 days of admission. Repeat TTE was performed 1 week after admission to reassess ventricular function. This examination revealed normal LV systolic function, but it demonstrated the new appearance of a vegetative mass on the posterior MV leaflet (Figures 1 and 2, Videos 1 and 2). An aortic vegetation was also visualized on the superior aspect of the noncoronary AV leaflet (Figure 3, Video 3). These findings could be suggestive of IE, but it was unlikely given the fact that the patient had been on broad-spectrum antibiotic coverage with cefepime and vancomycin since admission. The patient had also had negative bacterial and fungal blood cultures repeatedly throughout their hospitalization. Both ventricles demonstrated normal systolic function and no more than trace valvular regurgitation. Subsequent weekly echocardiography showed stable vegetation sizes and cardiac function, except for TTE about 1 month after hospitalization that showed mildly diminished LV systolic function (LVEF 48%), for which enalapril was started. Given the bleeding risk in the setting of leukemic coagulopathy and pulmonary hemorrhage, the patient was not a candidate for anticoagulation. As these complications were resolved, the patient was finally started on daily rivaroxaban 15 mg approximately 1 month after presentation. Despite the delay in the initiation of anticoagulation, serial echocardiography showed no increase in the size of the vegetations and no worsening of valvular function. Given the lack of valve dysfunction and the stability of vegetation size, surgical intervention was not indicated. The patient was extubated approximately 1 month after admission and was continued on the appropriate chemotherapy regimen. They were discharged to a rehabilitation facility approximately 2 months after presentation. Upon close follow-up with pediatric cardiology in clinic, serial echocardiographic studies demonstrated stable vegetation sizes, with normal valvular and ventricular function bilaterally.

## Discussion

LSE is an extremely rare condition in pediatric patients, and when reported, it is often associated with SLE. Eight cases of pediatric SLE-associated LSE have been documented in the literature.^1^ Malignancy-associated pediatric LSE appears to be even rarer. There was one reported case of malignancy-associated pediatric LSE in which an 8-year-old patient with high-grade non-Hodgkin’s lymphoma developed growing MV vegetations and progressive mitral regurgitation, prompting resection and confirmation of nonbacterial thrombotic endocarditis on histopathology.^8^

In our case, the combination of persistently negative blood cultures, inflammatory markers, and a new diagnosis of leukemia made malignancy-associated LSE the most probable diagnosis. Our patient underwent serial TTE weekly throughout their stay to continue monitoring cardiac function. The initial study showed mildly decreased LVEF, which prompted follow-up TTE 1 week later that showed valvular vegetations in the MV and AV. A study by Ali *et al*.^8^ noted rapid thickening of MV leaflets and progressive regurgitation in just a few days. However, as no repeat echocardiography was performed sooner than 1 week from the initial study in our patient, it is not possible to determine at what day the vegetations truly occurred. Our patient continued to have normal valvular function on multiple serial follow-up echocardiographic studies over the following 7 months from the initial date of diagnosis. In our case, surgical intervention was not indicated, as the vegetation size and valvular function remained stable on subsequent studies. Yet given the potential risk for rapid progression, close valvular function monitoring is warranted in these patients.

Distinguishing LSE from IE is critical because the management approaches are very different: IE requires prolonged antibiotic therapy, whereas LSE primarily demands anticoagulation to prevent embolic complications and immunosuppressive agents to dampen the inflammatory state associated with LSE.^9^ Prior studies have shown the efficacy of immunosuppressive agents, including corticosteroids, in treating valvular regurgitation, especially when initiated early in the disease course.^10^ In the setting of malignancy-associated LSE, management also focuses on treating the underlying malignancy. Our patient’s LSE was managed with anticoagulation and immunosuppressive agents (such as chemotherapeutic agents and systemic steroids), and surgical intervention was avoided given stable vegetation sizes and valvular function. Although systemic steroids were used as part of the leukemia treatment protocol in this case, their anti-inflammatory properties might have contributed to limiting vegetation progression and protecting against valvular dysfunction. Our hypothesis for the pathophysiology of malignancy-associated LSE is that the malignancy-induced prothrombotic state might have triggered the development of sterile valvular vegetations.^9^ However, underlying genetic susceptibility and specific malignancy subtypes may possibly be also contributing to its development.

This case underscores the importance of maintaining a broad differential when new valvular lesions are identified in patients with cancer. Prompt recognition of LSE allows the timely initiation of anticoagulation and immunosuppressive agents, reducing the risk for embolization, and prevents unnecessary use of antibiotics. Further studies might be warranted to evaluate efficacy of combined anticoagulation and systemic steroid therapy vs either anticoagulation or systemic steroids alone in the management of LSE.

## Conclusion

Even though LSE in pediatric patients has been mostly reported as a possible complication from existing SLE, the absence of autoimmune conditions should not rule out LSE, especially in the setting of malignancy, as was demonstrated in our case. Early diagnosis and appropriate anticoagulation and/or immunosuppressive therapy are key to preventing serious complications, avoiding unnecessary antibiotic exposure, and achieving better outcomes.

## Ethics Statement

The authors declare that the work described has been carried out in accordance with The Code of Ethics of the World Medical Association (Declaration of Helsinki) for experiments involving humans.

## Consent Statement

The authors declare that since this was a non-interventional, retrospective, observational study utilizing de-identified data, informed consent was not required from the patient under an IRB exemption status.

## Funding Statement

The authors declare that this report did not receive any specific grant from funding agencies in the public, commercial, or not-for-profit sectors.

## Disclosure Statement

The authors report no conflicts of interest.

## Supplementary Data

Video 1 Two-dimensional TTE, zoomed parasternal long-axis view, demonstrates an irregular, echogenic mass (*arrow*) attached to the atrial surface of the MV leaflet, consistent with a vegetation.

Video 2 Two-dimensional TTE, apical two-chamber view, demonstrates mildly reduced global LV systolic function with an irregular, echogenic mass (*arrow*) on the atrial surface of the posterior MV leaflet, consistent with a vegetation.

Video 3 Two-dimensional TTE, parasternal long-axis view, demonstrates a mobile, irregular, sessile, echogenic mass (*arrow*) attached to the ventricular surface of the noncoronary AV leaflet, consistent with a vegetation.
